# Real-world prognostic significance of attaining minimal residual disease negativity in newly diagnosed multiple myeloma

**DOI:** 10.1007/s12672-024-00891-8

**Published:** 2024-02-17

**Authors:** Jing Wang, Jing Li, Run Zhang, Jianyong Li, Lijuan Chen, Yuanyuan Jin

**Affiliations:** Department of Hematology, The First Affiliated Hospital of Nanjing Medical University, Jiangsu Province Hospital, No. 300 Guangzhou Road, Nanjing, 210029 China

**Keywords:** Multiple myeloma, Minimal residual disease, Prognostic factor

## Abstract

The aim of the study was to evaluate the prognostic impact of minimal residual disease (MRD) in the real-world setting and the interaction between MRD and molecular risk, clinical response and autologous stem-cell transplant (ASCT). A retrospective analysis of 275 newly diagnosed multiple myeloma (NDMM) patients who achieved very good partial remission (VGPR) or better before maintenance were involved. We examined MRD status by multiparameter flow cytometry (MFC). At a median follow-up of 37 months (4–88 months), In patients who achieved ≥ VGPR, those with MRD negativity had significantly longer PFS (51 *vs.* 26 months; *P* < 0.001) and OS (Not reached: NR *vs.* 62 months, *P* < 0.001) than those with MRD positivity. MRD positivity was the independent prognostic factor for PFS with hazard ratios of 2.650 (95% CI 1.755–4.033, *P* < 0.001) and OS with hazard ratios of 2.122 (95% CI 1.155–3.899, *P* = 0.015). Achieving MRD negativity was able to ameliorate a poor prognosis associated with genetic high risk. MRD negativity was associated with better PFS regardless of ASCT treatment. MRD status was more predictable for clinical outcome than conventional clinical responses. Moreover, Sustained MRD negativity ≥ 12 or ≥ 24 months improved both PFS and OS. Patients with NDMM who achieved MRD-negative status or sustained MRD negativity had deep remission and improved clinical outcomes regardless of high-risk cytogenetics, ASCT and clinical responses in a real-world setting.

## Introduction

Multiple myeloma (MM) is an incurable plasma cell disorder that usually produces monoclonal immunoglobulins (M-protein or monoclonal component). Therapeutic improvement over the last two decades made it possible for unprecedented response rates in myeloma patients leading to long-time survival. But even myeloma patients with such good responses still relapse due to the presence of residual tumor cells in the bone marrow. Conventional complete remission (CR) and subsequent attempts of stringent CR (sCR) were used to describe the depth of the response. The requirement of achieving deep responses with modern therapy led to the definition of negative minimal residual disease (MRD) which enable a higher prediction ability. MRD status has been shown a good surrogate marker for progression-free survival (PFS) and overall survival (OS) in a number of trials and meta-analyses [1–3]. The International Myeloma Working Group (IMWG) revised the MM response criteria in 2015 and introduced the definition of MRD in patients who have achieved a CR as an indication of long-term outcomes. However, results are conflicting regarding achievement of CR and minimal residual disease negativity. The percentage of patients with undetectable MRD showing positive immunofixation after induction was 22.5% by multiparameter flow cytometry(MFC), at a sensitivity level of ≤ 10^−4^ to 10^−5^ [4]. Even using next-generation sequencing(NGS), of which sensitivity was 10^–6^, the rates of discordance were 20% during treatment in the IFM-2009 clinical trial [5]. Besides, whether MRD negativity could overcome the initial molecular risk status and treatment remains controversial. IFM 2009 trial showed significantly longer PFS in those achieving MRD negativity irrespective of the treatment received (transplantation or VRD-only arms) or molecular risk status or ISS stage [5]. While in Myeloma XI, achieving MRD negativity was not able to overcome the adverse PFS associated with genetic high risk [6]. And meta-analysis confirmed that achieving MRD negativity in patients who had only achieved VGPR or better was associated with superior outcomes [2]. Sustained MRD negativity represents a deeper level of remission with a higher prognostic value [7, 8], especially lasting more than 1 year as IMWG recommended [9]. It remains unclear whether there are differences in clinical features and prognosis among MRD-negative patients who attain this status in different subgroups, such as those with high-risk cytogenetics, autologous stem cell transplantation (ASCT), or clinical response. In this article, we examined the MRD value in the real-world setting under different circumstances.

## Methods

### Patients

Patients undergoing consecutive therapy for NDMM at the Jiangsu Province Hospital between January 2015 and August 2022 were enrolled. Clinical data, including disease characteristics, the International Staging System (ISS) stage of disease, cytogenetic abnormalities, treatment specifics, disease response, and time to progression, were collected for analysis. High-risk cytogenetic abnormalities were defined as deletion 17p, t (4;14), t (14;16) as IMWG criteria using fluorescence in situ hybridization (FISH). The dosing, schedule, and regimen of induction received prior to ASCT varied between patients. Pre-ASCT high dose chemotherapy consisted of 200 mg/m^2^ or 140 mg/m^2^ melphalan according to creatinine clearance. Transplant-ineligible patients received at least 4 cycles induction and 4 cycles consolidation followed by maintenance. Response assessment was performed 3 months post-ASCT via serum and urinary protein electrophoresis, serum-free light chain assay, and bone marrow evaluation and defined according to the IMWG consensus criteria for response [10]. This study was approved by the Ethics Committee of the First Affiliated Hospital of Nanjing Medical University(No.2022-SR-448) and was conducted in accordance with the guidelines of the 1964 Declaration of Helsinki and its later amendments. Informed consents were obtained from all individual participants or their legal representatives or parents.

### MRD assessment

MFC MRD status was assessed in patients with a ≥ VGPR response at any time before maintenance therapy and 3 months after ASCT in transplant-eligible patients, and every 3 months during maintenance until progressive disease (PD). Patients provided specific written informed consent for MRD analyses. MFC analysis was performed in BM samples using at least 1 × 10^6^ cells, with the eight-color antibody combination: cLambda/CD56/CD138/CD27/cKappa/CD19/CD38/CD45. Immunophenotypic strategies for discriminating between normal and clonal plasma cells (PCs) have been described elsewhere [9]. MRD negativity was defined as < 10 clonal PCs detected by multiparameter flow cytometry after measuring ≥ 1 × 10^6^ nucleated cells, at a sensitivity level of 10^–5^. Data were acquired using a Navios flowcytometer (Beckman Coulter) and analyzed with Kaluza version 2.1 (Beckman Coulter) software.

### Statistical analysis

Statistical analyses were also performed with SPSS software (version 20.0; IBM, Chicago, IL). Baseline characteristics were evaluated using descriptive statistical analysis: frequency distributions (n, %) are presented for categorical variables and compared using Pearson’s chi-square test or Fisher’s test. The median (range) is presented for continuous variables and compared using a nonparametric test. PFS was the time between the date of diagnosis and the date of either the first documentation of progressive disease or death from any cause. OS was calculated from the date of diagnosis until death. PFS and OS were calculated by the Kaplan–Meier method. Multivariate analysis of variables associated with survival was conducted using the Cox Proportional-Hazard model for PFS and OS. *P* < 0.05 indicated that the difference was statistically significant.

## Results

### Patients and MRD assessments

**Fig. 1:**
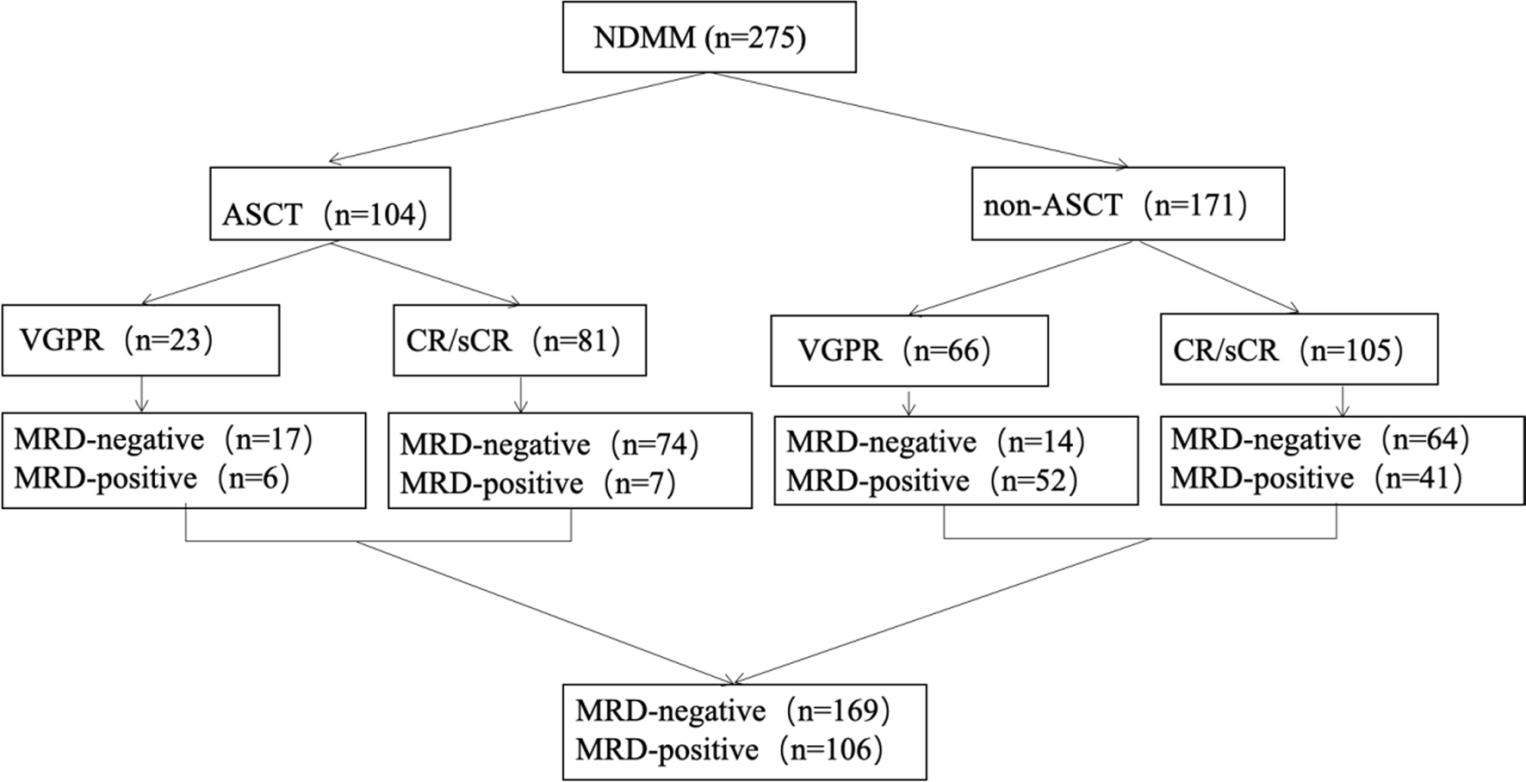
Study flow chart

275 consecutive patients with newly diagnosed MM (NDMM) achieved at least a VGPR before maintenance were assessed MRD (Fig. 1). In 104 patients underwent frontline ASCT, MRD status was negative in 17/23 (73.9%) in patients with VGPR, and 74/81 (91.3%) in those with CR respectively. In 171 transplant-ineligible patients, we observed MRD-negative in 14/66 (21.2%) VGPR patients, 64/105 (61%) in CR patients before maintenance (Fig. 1). In total, 169 were MRD-negative and 106 were MRD-positive, as defined by MFC. In 232 patients available for cIg-FISH(cytoplasmic light chain immunofluorescence with fluorescence in situ hybridization), 59(25.4%) were classified as having high-risk (HR) cytogenetics with del(17p) and/or t(4;14) and/or t(14;16) at diagnosis, others were divided into standard-risk (SR) group. Clinical characteristics and treatment compared between MRD-negative and MRD-positive patients were shown in Table 1. There were no significant differences between MRD-negative and MRD-positive patients in terms of gender, myeloma type, DS stage, ISS stage, BMPC ≥ 60%, LDH, calcium, albumin, renal function, hemoglobulin, cytogenetics, extramedullary diseases, circulating plasma cell ≥ 0.105. However, patients < 65 years had higher rate of MRD negativity attainment than those ≥ 65 years (*P* = 0.008). A total of 37.8% (104/275) patients were treated with ASCT which was more likely to achieve MRD negativity (*P* < 0.001). Patients achieved at least a CR also had higher percentage in achieving MRD negativity compared with VGPR ones(*P* < 0.001). While in multivariate analysis, age is not the sole determinant of MRD negativity (HR: 0.882, 95% CI 0.472–1.648, *P* = 0.695), patients treated with ASCT (HR: 0.192, 95% CI 0.672–0.351, *P* < 0.001) or achieved at least a CR (HR: 0.117, 95% CI 0.056–0.246, *P* < 0.001) had higher rate of MRD negativity attainment. MRD positivity was the strongest prognostic factor for progression-free survival with hazard ratios of 2.650 (95% CI 1.755–4.033, P < 0.001) (Fig. 4A).

**Table 1:** Patient and clinical characteristics

Clinical characteristics	MRD status, n (%)		*P* value
	MRD negative	MRD positive	
All	169 (61.5)	106 (38.5)	
Age (years)			0.008
≤ 65	122 (67.0)	60 (33.0)	
> 65	47 (50.5)	46 (49.5)	
Gender			0.911
Male	100 (61.1)	62 (38.9)	
Female	69 (61.7)	44 (38.3)	
Type of M protein			0.217
IgG	76 (59.8)	51 (40.2)	
IgA	38 (57.6)	28 (42.4)	
Light chain	43 (66.2)	22 (33.8)	
IgD	11 (84.6)	2 (15.4)	
IgM	1 (50.0)	1 (50.0)	
IgE	0 (0)	1 (100.0)	
Non-secretory	0 (0)	1 (100.0)	
DS stage			0.462
I	10 (71.4)	4 (28.6)	
II	29 (67.4)	14 (32.6)	
III	130 (59.6)	88 (40.4)	
ISS stage			0.124
I	39 (73.6)	14 (26.4)	
II	65 (59.6)	44 (40.4)	
III	65 (57.5)	48 (42.5)	
RISS stage (n = 258)	(n = 156)	(n = 102)	0.083
I	27 (77.1)	8 (22.9)	
II	105 (57.1)	79 (42.9)	
III	24 (61.5)	15 (38.5)	
Plasma cell (%)			0.443
< 60	156 (62.2)	95 (37.8)	
≥ 60	13 (54.2)	11 (45.8)	
Calcium (mmol/L)			0.964
≤ 2.75	147 (61.3)	92 (38.7)	
> 2.75	22 (62.0)	14 (38.0)	
Lactic dehydrogenase (IU/L)			0.626
normal	150 (62.0)	92 (38.0)	
elevated	19 (57.6)	14 (42.4)	
Albumin (g/L)			0.133
< 35	88 (57.5)	65 (42.5)	
≥ 35	81 (66.4)	41 (33.6)	
Creatinine (umol/L)			0.262
≤ 177	140 (63.1)	82 (36.9)	
> 177	29 (54.7)	24 (45.3)	
Hemoglobin (g/L)			0.266
< 100	97 (58.8)	68 (41.2)	
≥ 100	72 (65.5)	38 (34.5)	
Cytogenetics (n = 232)	(n = 141)	(n = 91)	0.791
Standard risk	106 (61.3)	67 (38.7)	
High risk	35 (59.3)	24 (40.7)	
1q21 amplification/gain			0.318
Positive	73 (64.0)	41 (36.0)	
Negative	68 (57.6)	50 (42.4)	
p53 deletion			0.796
Positive	14 (58.3)	10 (41.7)	
Negative	127 (61.1)	81 (38.9)	
T (4;14)			0.973
Positive	23 (60.5)	15 (39.5)	
Negative	118 (60.8)	76 (39.2)	
T (14;16)			1.000
Positive	1 (100.0)	0 (0)	
Negative	140 (60.6)	91 (39.4)	
Extramedullary diseases			0.680
With	37 (63.8)	21 (36.2)	
Without	132 (60.8)	85 (39.2)	
CPC (%) (n = 271)	(n = 168)	(n = 103)	0.209
< 0.105	126 (64.3)	70 (35.7)	
≥ 0.105	42 (56.0)	33 (44.0)	
ASCT			< 0.001
Yes	91 (87.5)	13 (12.5)	
No	78 (45.6)	93 (54.4)	
Response			< 0.001
VGPR	31 (34.8)	58 (65.2)	
CR/sCR	138 (74.2)	48 (25.8)	

### Impact of MRD status by molecular risk, treatment and response rates subgroups

**Fig. 2:**
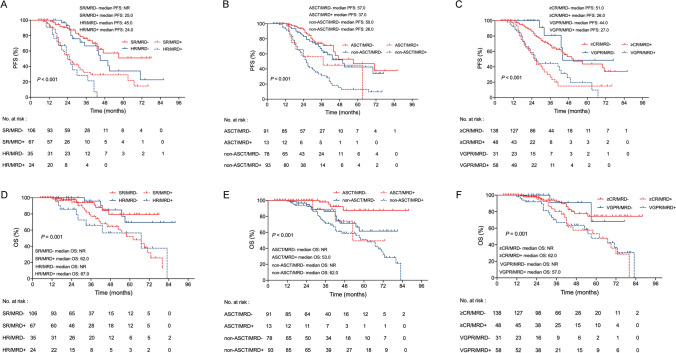
PFS (**A**) and OS (**D**) in MM patients according to combined cytogenetic risk and MRD status. PFS (**B**) and OS (**E**) in MM patients according to combined ASCT and MRD status. PFS (**C**) and OS (**F**) in MM patients according to combined clinical response and MRD status

To examine whether the MRD status would have prognostic impact on the baseline adverse cytogenetic properties defined by the IMWG, 232 patients with cytogenetic data were divided into four groups according to MRD status and cytogenetic risk stratification: Standard-risk and MRD-negative(SR/MRD-), High-risk and MRD-negative(HR/MRD-), SR/MRD + (Standard-risk and MRD-positive) and High-risk and MRD-positive(HR/MRD +). The HR/MRD- group had a similar median PFS and OS compared with SR/MRD- group (45 months vs. NR, *P* = 0.266; NR *vs.* NR, *P* = 0.921). Strikingly, the SR/MRD + group had a significantly shorter median PFS and OS compared with the HR/MRD- group (25 vs. 45 months, *P* = 0.006; 62 months vs. NR, *P* = 0.013). Suggesting that achieving MRD negativity was able to overcome the adverse PFS and OS associated with genetic high risk. MRD negativity was predictive of improved PFS and OS even in HR patients (Fig. 2A and D).

123 patients underwent induction regimens comprising bortezomib in combination with immunomodulatory drugs (IMiDs) including bortezomib, lenalidomide and dexamethasone (VRD), bortezomib, thalidomide and dexamethasone (VTD), bortezomib, pomalidomide and dexamethasone (VPD). Among them, 55% (68/123) underwent ASCT. 116 patients received bortezomib-based regimen including VCD (bortezomib, cyclophosphamide and dexamethasone), VD (bortezomib and dexamethasone) and VDD (bortezomib, liposome doxorubicin and dexamethasone), of which 27.5% (84/116) underwent ASCT. Additionally, 30 patients treated with IMiDs-based regimen, RD (lenalidomide and dexamethasone), BiRd (clarithromycin, lenalidomide and dexamethasone), CTD (cyclophosphamide, thalidomide and dexamethasone), of which 10% (3/30) underwent ASCT. 5 patients received daratumumab-based regimen and only 1 patient was treated with conventional chemotherapy. In the 171 transplant-ineligible patients, VRD (n = 38) and VCD (n = 60) were the most widely used regimens. The MRD-negative rate with bortezomib plus IMiDs induction followed by ASCT was 89.7%. The treatment group of bortezomib-based induction followed by ASCT had the second highest rate of MRD negativity at 87.5%.

Rates of MRD negativity were 87.5% (91/104) after ACST and 45.6% (78/171) without ASCT. ASCT had better PFS (57 vs. 34 months, *P* = 0.001) and superior OS (NR vs. 67 months, *P* < 0.001) than non-ASCT. Patients obtained MRD-negative after ASCT (ASCT/MRD- group) had similar PFS to those obtained MRD-negative before maintenance without undergoing ASCT (non-ASCT/MRD- group) (57 vs. 50 months, *P* = 0.330). However, ASCT/MRD- group showed significantly longer OS compared to non-ASCT/MRD- group (NR vs. NR, *P* = 0.011). No significant difference was found in PFS and OS between ASCT/MRD + and non-ASCT/MRD- groups (37 vs. 50 months, *P* = 0.165; 53 months vs. NR, *P* = 0.890). In transplant-eligible patients, post-transplant MRD negativity was associated with better PFS and better trends towards OS (*P* = 0.037; *P* = 0.057) compared to post-transplant MRD positivity. Meanwhile, in non-transplantation setting, this is even more of benefit (*P* < 0.001; *P* = 0.037) (Fig. 2B and E).

We also examined the relationship between MRD-negative attainment and clinical responses before maintenance. 81.7% (138/169) achieved MRD-negative status with ≥ CR, while 18.3% (31/169) achieved MRD-negative status with VGPR. Interestingly, patients who were still MRD-positive with ≥ CR had a significantly worse median PFS than MRD-negative with VGPR patients (26 vs. 44 months; *P* < 0.001). But only with trends for shortened OS (62 months vs. NR; *P* = 0.135). PFS and OS of patients in MRD-negative with CR are similar to that of patients achieving VGPR (51 vs. 44 months; *P* = 0.254; NR vs. NR; *P* = 0.080) (Fig. 2C and F). Together, these observations support that the MRD status might be more predictable for clinical outcome than conventional responses.

### Univariate and multivariate analyses of factors for PFS and OS

**Fig. 3:**
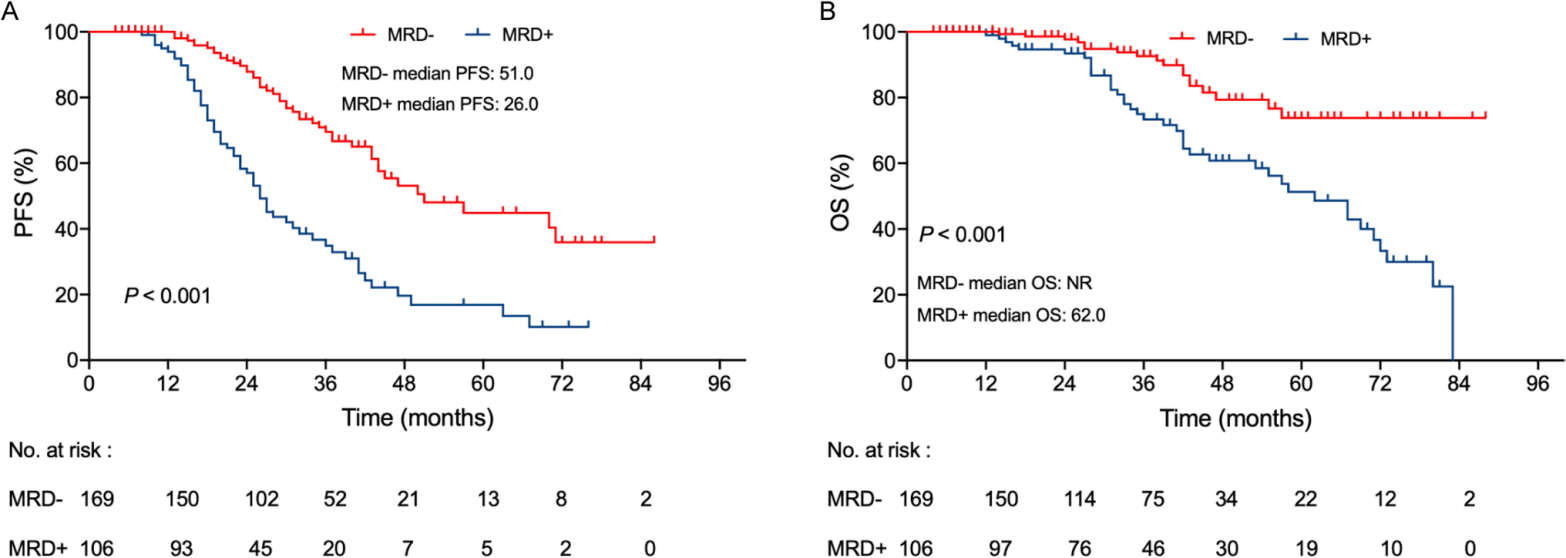
Kaplan-Meier survival curves for PFS (**A**) and OS (**B**) according to MRD status

At a median follow-up of 37 months (4–88 months), in patients who achieved ≥ VGPR, those with MRD negativity had significantly longer PFS (51 vs. 26 months; *P* < 0.001; Fig. 3A) and OS (NR vs. 62 months, *P* < 0.001; Fig. 3B) than their counterparts with MRD positivity.

**Fig. 4:**
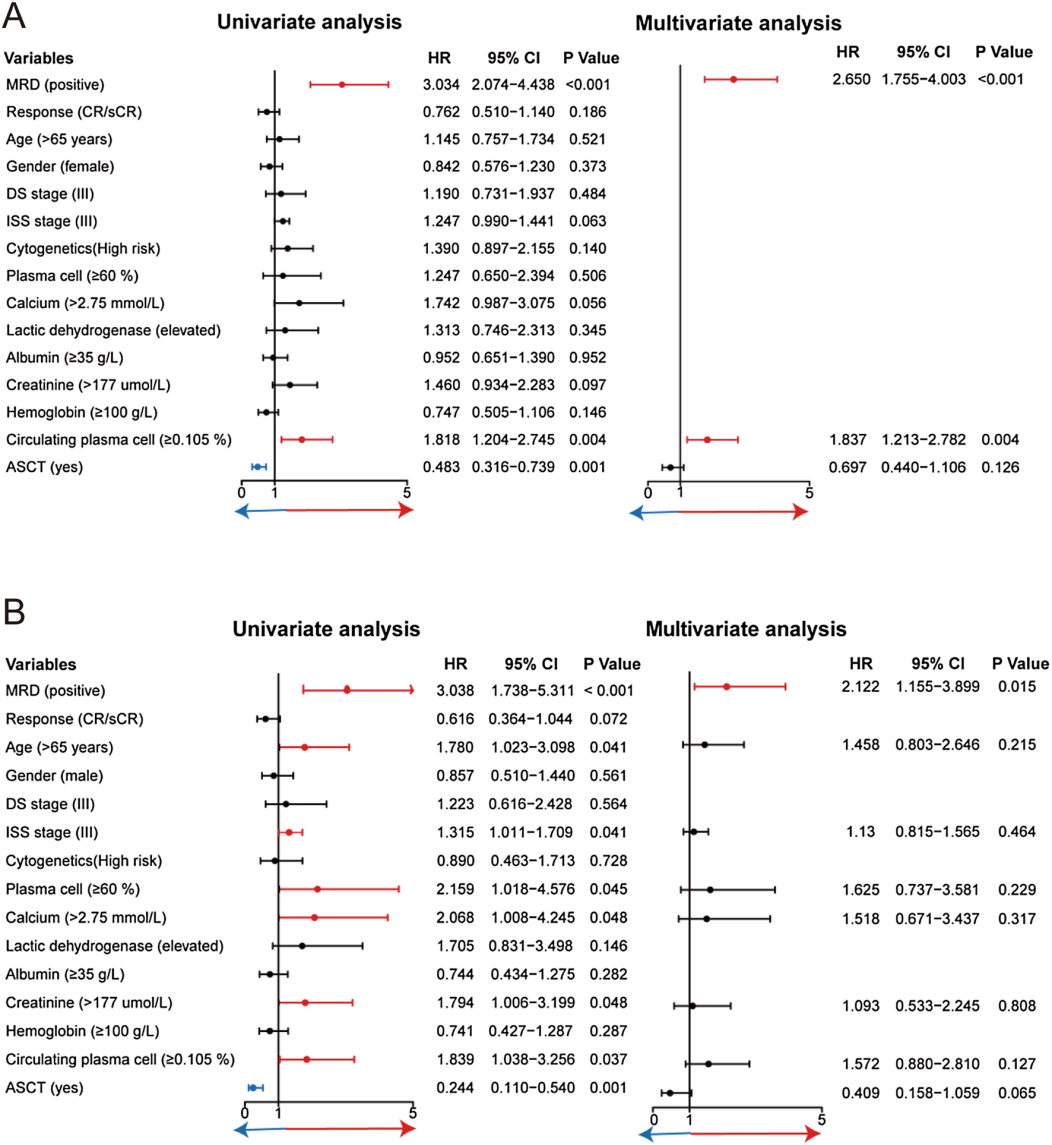
Univariate and multivariate Cox proportional hazards regressions for PFS (**A**) and OS (**B**)

In the univariate analysis, circulating plasma cell (≥ 0.105%), without ASCT treatment, and MRD positivity were associated with shorter PFS, while hypercalcemia and ISS III stage showed trends. In multivariate Cox models, MRD positivity was the strongest prognostic factor for progression-free survival with hazard ratios of 2.650 (95% CI 1.755–4.033, *P* < 0.001) (Fig. 4A).

For OS, elderly patients (age ≥ 65 years), ISS stage III, renal insufficiency, hypercalcemia, bone marrow plasma cell ≥ 60%, circulating plasma cell ≥ 0.105%, without ASCT treatment, and MRD positivity had negative impact. But only MRD positivity has the independent prognostic value for OS with hazard ratios of 2.122 (95% CI 1.155–3.899, *P* = 0.015) (Fig. 4B).

### Impact of MRD-negative durability

**Fig. 5:**
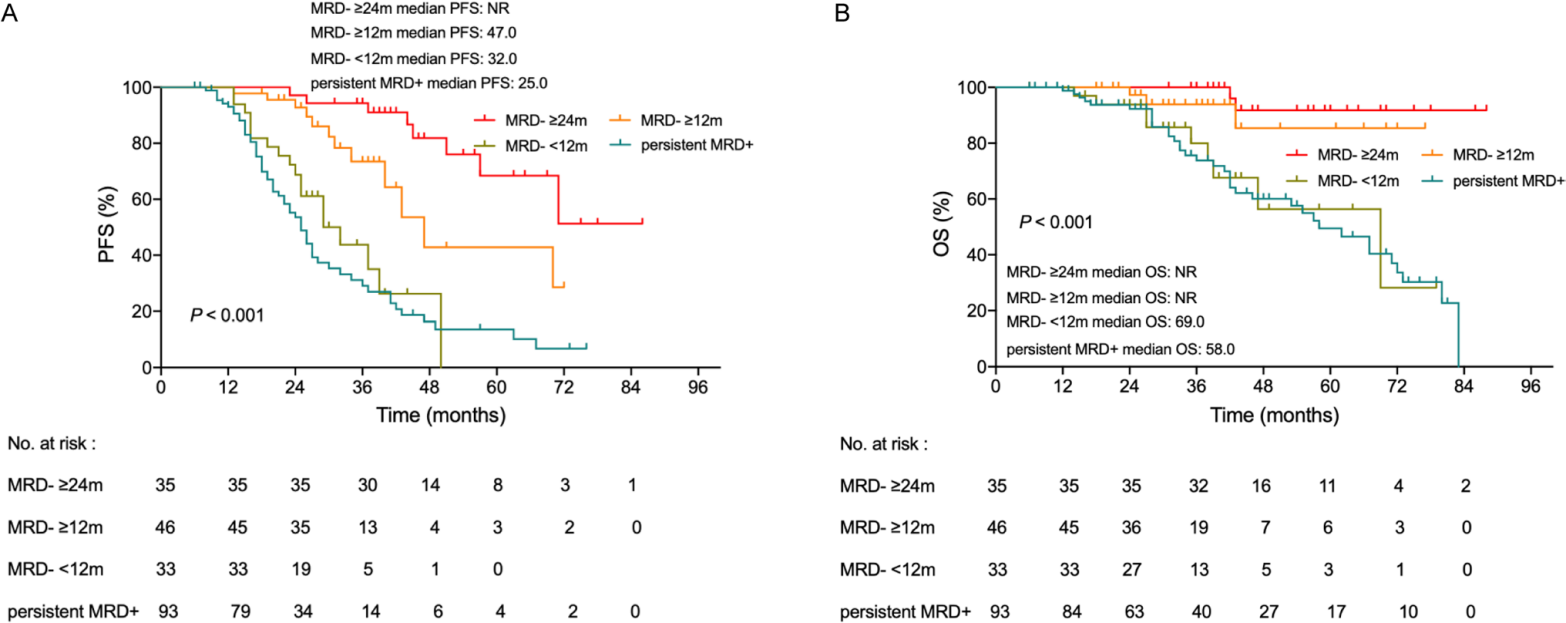
Kaplan-Meier survival curves for PFS (**A**) and OS (**B**) according to sustained MRD negativity≥12 months and ≥24 months

MRD durability was assessed among patients achieving ≥ 2 MRD-negative results lasting ≥ 12 or ≥ 24 months with no MRD-positive result in between. Consecutive MRD data were available for 207 patients while 68 MRD subsequent data were missing. 93 of 103 MRD-positive patients harbored sustained MRD positivity during maintenance. The remaining 10 patients converted to MRD-negative patients during maintenance some of whom also lasted for ≥ 12 months. In patients achieving MRD negativity(MRD-) before maintenance. 44 patients achieved sustained MRD negativity lasting ≥ 12 months, 33 patients achieved sustained MRD negativity lasting ≥ 24 months, the rest of 27 patients did not achieve ≥ 12 months MRD negativity. Eventually, 46 patients with sustained MRD negativity lasting ≥ 12 months, 35 patients with sustained MRD negativity lasting ≥ 24 months, 33 patients with < 12 months MRD negativity and 93 patients with sustained MRD positivity(MRD +) were divided into 4 groups for analysis. PFS was prolonged in patients with sustained MRD-negative durability lasting ≥ 12 months or ≥ 24 months compared with patients who did not achieve sustained MRD negativity or patients who were MRD-positive (MRD- ≥ 12 months vs. MRD- < 12 months or MRD + ; 47 vs. 32 or 25 months;* P* = 0.001, *P* < 0.001; MRD- ≥ 24 months vs. MRD- < 12 months or MRD + ; NR *vs.* 32 or 31 months; *P* < 0.001, *P* < 0.001). Meanwhile, the prolonged OS were also shown in patients with sustained MRD negativity compared with those with MRD negativity < 12 months or MRD positivity (MRD- ≥ 12 months vs. MRD- < 12 months or MRD + ; NR vs. 69 or 58 months; *P* = 0.025,* P* = 0.006; MRD- ≥ 24 months vs. MRD- < 12 months or MRD + ; NR vs. 69 or 58 months; *P* = 0.001, *P* < 0.001). These data supported that MRD negativity lasting ≥ 12 or ≥ 24 months are each associated with improved PFS and OS (Fig. 5). PFS and OS only didn’t show trends for improving between patients with persistent MRD positivity compared to those with MRD negativity < 12 months (25 vs. 32 months, *P* = 0.229; 58 vs. 69 months, *P* = 0.834). However, the durability of sustained MRD negativity also matters, MRD-negative duration ≥ 24 months had significantly superior PFS compared with MRD-negative duration ≥ 12 months (NR vs. 47 months, *P* = 0.021). The two group didn’t show difference in OS (NR *vs.* NR, *P* = 0.320).

## Discussion

CR by this conventional definition was not precise enough to reflect disease control, subsequent attempts to improve response assessment using MRD negativity may be a more robust evaluation of disease control in the novel agent era. MRD evaluation is now a fundamental surrogate for survival in NDMM for redefining the goal of therapy, focusing on long-term control, quality of life, and even a cure [11]. We found higher rates of MRD negativity among patients who underwent transplantation than among those who didn’t. Moreover, the value of CR is intrinsically related to a high proportion of patients who were MRD-negative. Age was found to be a contributing factor with respect to achieving an MRD-negative status. Age < 65 years, clinical response ≥ CR and ASCT were associated with achieving an MRD-negative status.

In our study, we confirmed the prognostic role of MRD negativity in a large cohort of NDMM patients with ≥ VGPR status. Our results are consistent with previous publications showing that MRD negativity is associated with improved PFS and OS for multiple myeloma and also was found to confer a more than 50% relative risk reduction in both progression and mortality [2, 12]. Besides, we also explored the applicability of these findings to various subgroups of patients with MM. PFS and OS were prolonged in both high- and standard-risk myeloma patients who were MRD-negative than in those who are MRD-positive. This result was consistent with those presented in previous studies [13–16]. However, whether achieving MRD negativity can thoroughly ameliorate adverse risk factors identified at diagnosis? Our results showed that compared to standard-risk myeloma patients with MRD negativity, high-risk myeloma patients with MRD negativity showed similar survival outcome. Thus, achieving MRD negativity may be associated with superior outcomes in patients even with high-risk cytogenetics. Furthermore, we also explore the MRD impact in the transplantation and non-transplantation subgroups. The best PFS and OS were seen in post-ASCT MRD-negative patients. OS in MRD-negative patients in the transplantation group was superior to that in patients in the non-transplantation group with the same MRD status. Of note, in cases where MRD negativity was not attained after ASCT, the survival outcomes were comparable to those who did not undergo ASCT. It seems possible that MRD monitoring could identify patients who might benefit from ASCT. Further intensify therapy may be added in post-transplantation MRD-positive patients. Moreover, patients who were MRD-negative despite persistent M component showed PFS and overall survival were as favorable as those of MRD-negative patients in CR. Similar findings were reported in the clinical trials [17–19]. This unexpected discordance may be driven by long half live of immunoglobulins or oligoclonal responses after treatment [4, 20, 21].

Our study has shown that MRD status is a robust and independent prognostic indicator for both PFS and OS, with particular significance observed in the OS analysis, where it was the only prognostic marker found to be significant in multivariate analysis. Interestingly, our study did not find high-risk cytogenetics or ASCT, which are typically considered to be important factors, to be significant predictors of survival outcomes. In 2016, IMWG recommended the concept of sustained MRD negativity, defined as MRD negativity lasting for a duration of more than 1 year [9]. Our current analysis demonstrated that patients with sustained MRD negativity lasting either ≥ 12 or ≥ 24 months had a longer time to progression and death. Sustained MRD negativity lasting ≥ 24 months was even associated with a longer PFS than lasting for ≥ 12 months. Clinical decisions such as discontinuation, intensification or initiation of a new therapy based on measurable residual disease treatment were confirmed to improve PFS [22]. Thus, more clinical trials might bring us the endpoint of maintenance [23].

## Conclusion

With the advances in therapy targeting the myeloma cell, achieving minimal residual disease states is now possible. Data clearly support the robust evaluation of disease control if sustained over time, with patients achieving an MRD-negative status having improved PFS and OS. Consequently, our findings suggest that MRD negativity can be a valuable endpoint in informing treatment decisions and providing significant reassurance for myeloma patients who achieve this status, regardless of their cytogenetic risk or previous treatment or clinical response.

## Data Availability

The data are available from the authors upon reasonable request.
